# Synthesis and Evaluation of Selected Benzimidazole Derivatives as Potential Antimicrobial Agents

**DOI:** 10.3390/molecules200815206

**Published:** 2015-08-20

**Authors:** Fatmah A. S. Alasmary, Anna M. Snelling, Mohammed E. Zain, Ahmed M. Alafeefy, Amani S. Awaad, Nazira Karodia

**Affiliations:** 1 Chemistry Department, College of Science, King Saud University, Riyadh 11362, Saudi Arabia; 2 Centre for Pharmaceutical Engineering Science, Faculty of Life Sciences, University of Bradford, Richmond Road, Bradford BD7 1DP, UK; E-Mail: a.m.snelling@bradford.ac.uk; 3 Botany and Microbiology Department, College of Science, King Saud University, Riyadh 11362, Saudi Arabia; E-Mail: mohamedzain@hotmail.com; 4 Department of Pharmaceutical Chemistry, College of Pharmacy, Sattam bin Abdulaziz University, Al-Kharj 11942, Saudi Arabia; E-Mail: alafeefy@hotmail.com; 5 Pharmacognosy Department, College of Pharmacy, Salman bin Abdulaziz University, Al-Kharj 11942, Saudi Arabia; E-Mail: amaniawaad@hotmail.com; 6 Faculty of Science and Engineering, University of Wolverhampton; Wulfruna Street, Wolverhampton WV1 1LY, UK

**Keywords:** benzimidazole, heterocycle, antibacterial activity, antifungal activity, resistance, Gram-negative, Gram-positive

## Abstract

A library of 53 benzimidazole derivatives, with substituents at positions 1, 2 and 5, were synthesized and screened against a series of reference strains of bacteria and fungi of medical relevance. The SAR analyses of the most promising results showed that the antimicrobial activity of the compounds depended on the substituents attached to the bicyclic heterocycle. In particular, some compounds displayed antibacterial activity against two methicillin-resistant *Staphylococcus aureus* (MRSA) strains with minimum inhibitory concentrations (MICs) comparable to the widely-used drug ciprofloxacin. The compounds have some common features; three possess 5-halo substituents; two are derivatives of (*S*)-2-ethanaminebenzimidazole; and the others are derivatives of one 2-(chloromethyl)-1*H*-benzo[*d*]imidazole and (1*H*-benzo[*d*]imidazol-2-yl)methanethiol. The results from the antifungal screening were also very interesting: 23 compounds exhibited potent fungicidal activity against the selected fungal strains. They displayed equivalent or greater potency in their MIC values than amphotericin B. The 5-halobenzimidazole derivatives could be considered promising broad-spectrum antimicrobial candidates that deserve further study for potential therapeutic applications.

## 1. Introduction

Microbial drug resistance is a serious issue, especially as increasing numbers of strains are becoming resistant to multiple antimicrobial agents, with some bacteria now being resistant to all available antibiotics. There is thus a critical need to develop new drugs with novel mechanisms of action. However, the investment available for such development is frequently lower than the required level. The development of new drug entities is hampered by several issues, notably the high cost and length of time required, as well as the logistical and regulatory challenges of performing the necessary clinical evaluations across multiple geographical areas. Therefore, a few new classes of antimicrobials have been developed since the late 1980s [1,2,3], and much research has focused only on the chemical modification of existing drugs to improve their potency and/or ability to overcome antibiotic resistance mechanisms. Even if this approach does not improve antimicrobial activity directly, it may lead to derivatives that can usefully inhibit virulence mechanisms [4].

Compounds having benzimidazole as a structural motif have been widely used in medicinal chemistry and drug development, and researchers are actively seeking new uses and applications of this heterocycle [5]. Benzimidazole-containing compounds have numerous medical and biological activities, such as antitumor [6] antibacterial [7,8,9,10], antifungal [11], antiviral [12,13,14,15,16], anticonvulsant [17], antidepressant [18], analgesic [19], anti-inflammatory[20] and antidiabetic properties [21]. For example, derivatives, such as thiabendazole, cambendazole, parbendazole, mebendazole, albendazole and flubendazole, are widely-used anti-helminth drugs, used to treat people and animals with gastrointestinal worm infections [22]. Two groups of benzimidazole derivatives, namely 5,6-dinitro- and 2-trifluromethyl derivatives, are particularly well known for their use as antihelminth drugs [23]. 2-Methoxycarbonylamino derivatives have shown good antiprotozoal activities against some protozoan parasites, such as *Giardia lamblia* and *Entamoeba histolytica*, by inhibiting tubulin polymerization, and hence, making these better antiprotozoal agents than metronidazole and albendazole [24]. Nitrogen-containing heterocyclic systems have a diverse spectrum of pharmacological properties. Different heterocyclic motifs can be incorporated to produce molecules with enhanced biological properties. Recent reports include benzimidazoles bearing the 1,3,4-oxadiazole moiety, which have broad spectrum antimicrobial properties [25], and molecules containing both the benzimidazole and indole heterocycles, which exhibit selective antibacterial activity [26].

A review of the literature thus suggests that there is the scope for the design of additional benzimidazole derivatives with antimicrobial activity, by examining the effect of a number of different functional groups.

In this paper, we report on the synthesis of a series of benzimidazole derivatives and their antimicrobial activity. A detailed study of the structure-activity relationship of these derivatives will pave the road to designing more potent compounds. The compounds were tested for their ability to inhibit isolates amongst a reference panel of 26 bacterial and 10 fungal strains, and key results are presented in Table 1, Table 2 and Table 3.

**Table 1:** Benzimidazole derivatives synthesized.

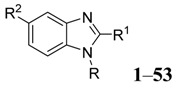

Compound	R	R^1^	R^2^
**1**	H	H	H
**2**	H	H	CH_3_
**3**	H	H	OCH_3_
**4**	H	H	Cl
**5**	H	H	Br
**6**	H	H	F
**7**	H	H	NO_2_
**8**	H	H	CN
**9**	H	CH_2_NH_2_	H
**10**	H	CH_2_NH_2_	CH_3_
**11**	H	CH_2_NH_2_	Cl
**12**	H	CH_2_NH_2_	Br
**13**	H	CH_2_NH_2_	F
**14**	H	CH_2_NH_2_	NO_2_
**15**	H	CH(CH_3_)NH_2_	H
**16**	H	CH(CH_3_)NH_2_	CH_3_
**17**	H	CH(CH_3_)NH_2_	Cl
**18**	H	CH(CH_3_)NH_2_	Br
**19**	H	CH(CH_3_)NH_2_	F
**20**	H	CH_2_CH_3_	H
**21**	H	CH_2_CH_3_	NO_2_
**22**	H	CH_2_SH	H
**23**	H	CH_2_SH	NO_2_
**24**	H	CH_2_OH	H
**25**	H	CH_2_OH	CH_3_
**26**	H	CH_2_OH	OCH_3_
**27**	H	CH_2_OH	Cl
**28**	H	CH_2_OH	Br
**29**	H	CH_2_OH	F
**30**	H	CH_2_OH	NO_2_
**31**	H	CH_2_OH	CN
**32**	H	CH_2_Cl	H
**33**	H	CH_2_Cl	CH_3_
**34**	H	CH_2_Cl	OCH_3_
**35**	H	CH_2_Cl	Cl
**36**	H	CH_2_Cl	Br
**37**	H	CH_2_Cl	F
**38**	H	CH_2_Cl	NO_2_
**39**	H	CH_2_Cl	CN
**40**	H	COOH	H
**41**	H	COOH	CH_3_
**42**	H	COOH	OCH_3_
**43**	H	COOH	Cl
**44**	H	COOH	Br
**45**	H	COOH	F
**46**	H	COOH	NO_2_
**47**	CH_3_	CH_2_OH	H
**48**	CH_3_	CH_2_OH	CH_3_
**49**	CH_3_	CH_2_OH	Cl
**50**	CH_3_	CH_2_OH	Br
**51**	CH_3_	CH_2_OH	F
**52**	CH_3_	CH_2_OH	OCH_3_
**53**	CH_3_	CH_2_OH	NO_2_

## 2. Results and Discussion

### 2.1. Chemistry

**Figure 1:**
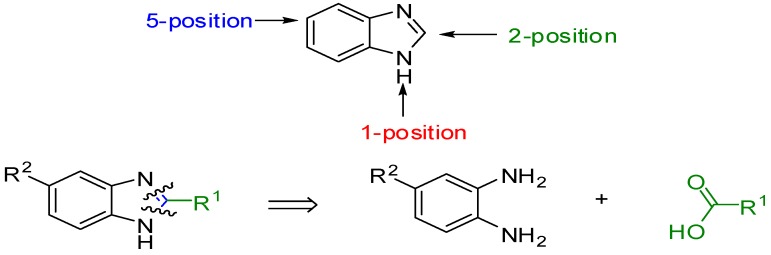
Retrosynthetic analysis of benzimidazole.

**Scheme 1:**
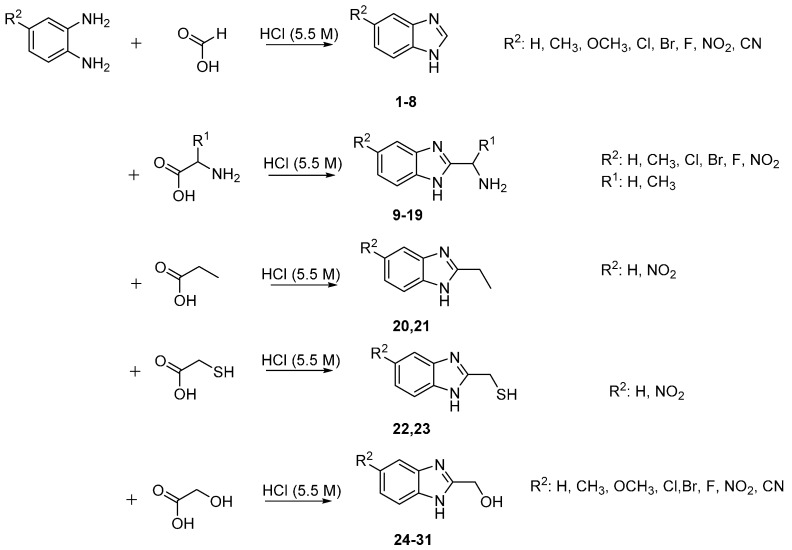
2,5-Substituted benzimidazole derivatives.

Our target molecules were based on benzimidazole derivatives. It is possible to design a wide range of potential microbial inhibitors by replacing the hydrogen at various positions of the benzimidazole ring with different functional groups. However, the most accessible derivatives are those with substituents at the 1-, 2- and 5-positions. Retrosynthetic analysis of a 2,5-disubstituted benzimidazole identified two fragments, which explains why these particular substituted benzimidazoles are easy to prepare (Figure 1). The reactions of substituted 1,2-phenylenediamines and carboxylic acids can provide access to a library of compounds. In this application, the design of these inhibitors focused on 5-substituted benzimidazoles followed by the synthesis of 2,5-disubstituted analogues based on (1*H*-benzimidazole-2-yl)methanol derivatives, (1*H*-benzimidazole-2-yl)alkylamines derivatives, (1*H*-benzimidazole-2-yl)-ethyl derivatives and (1*H*-benzimidazole-2-yl)-methanethiol derivatives (Scheme 1). The general method for the synthesis of these benzimidazoles **1**–**31** was based on the Phillips procedure [27].

**Scheme 2:**
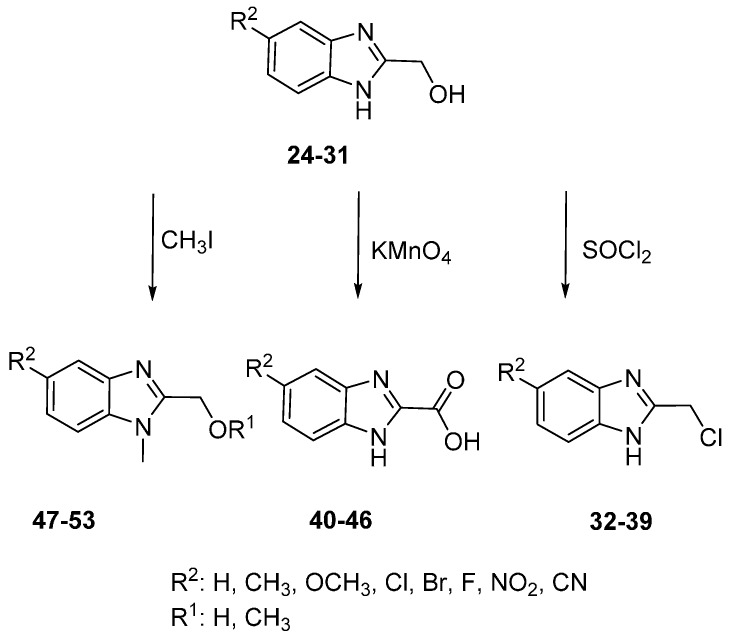
Conversion of 2-methanol benzimidazole derivatives.

The 1*H*-benzimidazole-2-yl)methanol derivatives were readily reacted further (oxidation, chlorination and *N-*alkylation) (Scheme 2). The hydroxyl group of (1*H*-benzimidazole-2-yl)methanol was converted into the chloromethyl through a reaction with thionyl chloride to give 2-(chloromethyl)-1*H*-benzimidazole Derivatives **32**–**39**. The 2-chloromethylbenzimidazoless were required for the biological studies to provide a direct comparison with other functional groups in the 2-position of benzimidazole [28]. Benzimidazole-2-carboxylic Acids **40**–**46** were readily obtained through simple oxidation of (1*H*-benzimidazole-2-yl)-methanol Derivatives **24**–**30** using potassium permanganate. The *N-*methylation of Derivatives **24**–**30** gave the corresponding *N*-methyl-5-substituted (1*H*-benzimidazole-2-yl)methanol derivatives **47**–**53**.

### 2.2. Biological Evaluation

#### 2.2.1. Antibacterial Activity

Firstly, a cut-off point of an inhibition zone diameter of ≥12 mm was used to define appreciable antibacterial activity. Using this criteria, disc diffusion tests with the 53 compounds revealed that nine compounds (**9**, **17**, **18**, **22**, **32**, **33**, **35**, **36** and **52**) had some degree of antibacterial activity (Table 2); with five exhibiting activity against representatives of at least two *Staphylococcal* species. Six compounds had activity against one or more strains of Methicillin-resistant *Staphylococcus aureus* (MRSA), including strains that were resistant to ciprofloxacin. Six compounds had some activity against *S. epidermidis* and three against the single strain of *S. haemolyticus* used in this study. Regarding the Gram-negative bacteria, only two (**32** and **52**) of the compounds tested were active against any of the five strains of *Escherichia coli* and none against the four strains of *Pseudomonas aeruginosa*. Two compounds were active against the single strain of *Serratia marcescens*, but six had some activity against the single strain of *B. cepacia* used in the screening panel. Compounds **17**, **18**, **22**, **35** and **36** were the most active compounds overall, and these were selected on the basis of a broad spectrum of activity, and/or wide zones of inhibition, or novel chemical structure. As summarised in Table 2, these five compounds were investigated further in minimum inhibitory concentration (MIC) assays to quantify their activity against the reference isolates. Testing confirmed that they exhibited little activity against the Gram-negative species, with the exception of *Burkholderia cepacia*. However, for the Gram-positive species, there were some MICs as low as 64 and even 32 µg/mL. Notably, Compounds **17** and **18** had MICs comparable to that of ciprofloxacin against two of the MRSA strains, whilst **22** and **36** had consistent activity (MIC 32–64 µg/mL) against almost all of the staphylococci. This is significant and provides lead compounds for further development.

**Table 2:** Antibacterial activity of selected benzimidazole derivatives against Gram-positive and Gram-negative bacteria (series of 2-ethanamine, 2-methanthiol, 2-chlromethyl and *N*-methyl benzimidazole derivatives).

Bacterial Species Compound	Diameter of the Inhibition Zone (mm) around Each Compound in the Agar Diffusion Test									
	9	17	18	22	32	33	35	36	52	Ciprofloxacin
**Gram-Positive**										
*Staphylococcus aureus* NCTC 6571	0	10	11	11	**13**	9	9	9	**14**	**27**
*Staphylococcus aureus* NCTC 10399	0	**12**	**12**	**16**	**7**	7	0	0	**17**	**33**
MRSA HG-1	0	10	11	**12**	0	0	0	**13**	**13**	0
MRSA-15 NCTC 13142	0	11	10	**12**	0	0	9	**14**	**15**	**28**
MRSA-16 NCTC 13143	0	10	**13**	**15**	0	0	10	**15**	**13**	0
MRSA BIG 0043	0	11	10	11	9	10	9	11	**12**	0
MRSA BIG 0044	0	9	**12**	**12**	10	10	9	**15**	**12**	0
MRSA BIG 0045	0	11	11	**14**	0	**12**	9	11	**13**	0
MRSA BIG 0047	0	**12**	**13**	**12**	0	11	9	11	**13**	0
MRSA BIG 0050	0	0	**12**	11	0	9	9	**15**	**13**	0
MRSA BIG 0052	0	0	**13**	11	0	9	9	**14**	**12**	0
MRSA BIG 0053	0	0	11	**13**	0	10	9	**14**	**13**	0
*Staphylococcus epidermidis* NCTC 11047	0	0	11	0	10	**12**	0	0	**12**	**32**
*Staphylococcus epidermidis* NCTC 2749	**17**	9	**13**	**13**	9	10	**14**	9	**13**	**35**
*Staphylococcus haemolyticus* NCTC 11042	0	**12**	10	**18**	11	11	0	0	**15**	**34**
**Gram-Negative**										
*Burkholderia cepacia* NCTC 10744	**12**	11	10	**15**	0	**12**	**15**	**17**	**12**	**27**
*Escherichia coli* NCTC 10418	0	0	0	0	**13**	9	9	9	**14**	**33**
*Escherichia coli* BIG 0046	0	0	0	0	9	9	8	0	9	0
*Escherichia coli* BIG 0048	0	0	0	0	**15**	9	8	8	11	0
*Escherichia coli* BIG 0049	0	0	0	0	11	0	7	8	8	0
*Escherichia coli* 0051	0	0	0	0	**12**	9	7	0	10	0
*Pseudomonas aeruginosa* NCTC 6749	0	0	0	0	10	0	0	0	10	**33**
*Pseudomonas aeruginosa* BIG 0039	0	0	0	0	0	0	0	0	8	**34**
*Pseudomonas aeruginosa* NCTC 10662	0	0	0	0	10	0	0	0	8	**32**
*Pseudomonas aeruginosa* BIG 0063	0	0	0	0	10	0	0	0	9	**30**
*Serratia marcescens* NCTC 1377	0	0	0	0	**15**	8	9	8	**13**	**32**

Zone diameters indicative of antibacterial activity (*i.e.*, ≥12 mm) are highlighted in bold.

Interestingly, the MICs of Compounds **17** and **18** (2-ethanamine benzimidazole derivatives) against the investigated bacteria were similar to each other (Table 3). This suggests that the higher electronegativity of bromine and chlorine increased the antibacterial activity, and this was also observed by Tavman [29].

**Table 3:** MICs of the selected benzimidazole derivatives against the reference panel of Gram positive and Gram negative bacteria.

Bacterial Strain	MIC (µg/mL) per Compound					
	17	18	22	35	36	Ciprofloxacin
**Gram-Positive**						
*Staphylococcus aureus* NCTC 6571	**32**	**32**	64	128	64	≤0.5
*Staphylococcus aureus* NCTC 10399	256	256	64	128	64	>32
MRSA HG-1	**32**	**32**	64	265	64	**32**
MRSA-15 NCTC 13142	256	256	64	128	64	≤0.5
MRSA-16 NCTC 13143	256	256	64	256	64	32
MRSA BIG 0043	256	256	64	256	64	>32
MRSA BIG 0044	>512	>512	64	256	64	>32
MRSA BIG 0045	256	256	64	256	>512	8
MRSA BIG 0047	256	256	64	256	64	8
MRSA BIG 0050	**32**	**32**	64	256	64	**32**
MRSA BIG 0052	256	256	64	256	64	≤0.5
MRSA BIG 0053	256	256	64	256	64	>32
*Staphylococcus epidermidis* NCTC 11047	256	256	32	256	32	≤0.5
*Staphylococcus epidermidis* NCTC 2749	256	256	64	256	64	≤0.5
*Staphylococcus haemolyticus* NCTC 11042	256	265	32	256	32	8
**Gram-Negative**						
*Burkholderia cepacia* NCTC 10744	32	32	64	256	64	≤0.5
*Escherichia coli* NCTC 10418	>512	>512	512	>512	>512	≤0.5
*Escherichia coli* BIG 0046	>512	>512	>512	>512	>512	32
*Escherichia coli* BIG 0048	>512	>512	512	>512	>512	8
*Escherichia coli* BIG 0049	>512	>512	>512	>512	>512	8
*Escherichia coli* BIG 0051	>512	>512	>512	>512	>512	32
*Pseudomonas aeruginosa* NCTC 6749	>512	>512	>512	512	265	≤0.5
*Pseudomonas aeruginosa* BIG 0039	>512	>512	>512	512	512	≤0.5
*Pseudomonas aeruginosa* NCTC 10662	>512	>512	>512	512	512	≤0.5
*Pseudomonas aeruginosa* BIG 0063	>512	>512	>512	512	512	≤0.5
*Serratia marcescens* NCTC 1377	>512	>512	>512	>512	>512	≤0.5

Good activity, relative to the MIC of ciprofloxacin, is indicated in bold. The results are the average of three independent readings.

For the 2-methanthiol benzimidazole derivatives (**22**–**23**, Scheme 1), the unsubstituted derivative at position 5, Compound **22**, was selectively active against all of the Gram-positive bacteria with an MIC of 32 µg/mL against *S. epidermidis* NCTC 11047 and *S. haemolyticus* NCTC 11042 and MIC 64 µg/m against the other staphylococci plus *B. cepacia* (Table 3). The inhibitory activity decreases when position 5 is methylated as in Compound **23** (>512 MIC µg/mL). This was also observed by Podunavac-Kuzmanovic and Cvetkovic [30], who found that the unsubstituted analogue was more potent than the methyl analogues of the 2-aminobenzimidazole against strains of *Bacillus cereus*, *Staphylococcus aureus*, *Sarcina lutea* and *Pseudomonas aeruginosa*.

From the series of 2-chloromethyl benzimidazole derivatives (Scheme 2), **36** (5-Br) was the most active compound with inhibition zone diameters of 13–17 mm. The largest zones of inhibition (17 mm and 15 mm diameter) were exhibited by Compounds **35** (5-Cl) and **36**, respectively, against *Burkholderia cepacia* NCTC 10744. Only **35** and **36** were capable of inhibiting some strains at a concentration below 256 μg/mL. As shown in Table 3, Compound **36**, the brominated derivative, was more active than Compound **35**, which is the chlorinated derivative. This could support the hypothesis that there is a direct relationship between biological activity and the electron withdrawing effect [29].

#### 2.2.2. Antifungal Activity

The well diffusion tests of the 53 compounds against the fungi revealed very interesting results: 21 compounds were active against *Aspergillus clavatus* RCMB 2593; 28 had some activity against *Aspergillus fumigatus* RCMB 02564; 26 compounds were active against *Penicillium marneffei* RCMB 01267; 22 compounds were active against *Mucor circinelloides* RCMB 07328; 20 compounds were active against *Absidia corymbifera* RCMB 09635; 20 compounds were active against *Syncephalastrum racemosum* RCMB 05922; 29 compounds were active against *Candida albicans* RCMB 05035; 29 compounds were active against *Candida tropicalis* RCMB 05049; 27 compounds were active against *Candida parapsilosis* RCMB 05065; 13 compounds had some activity against *Candida krusei* RCMB 05051; 14 compounds had no detectable antifungal activity.

Amphotericin B was used as a positive control for the antifungal testing. The intention was not to compare activity on a molar basis, since such a comparison does not necessarily have biological validity when comparing compounds with very different modes of action or solubility in diffusion assays. This allowed us to identify the compounds with the most promising activity, which could be looked at in more detail in further studies.

Table 4 and Table 5 summarise the results for the 23 compounds that were most active against the fungal strains used. The antifungal screening of the series of 2-ethanamine benzimidazole Derivatives **9**–**19** showed remarkable antifungal activities against both unicellular and filamentous fungi (Table 4). However, Compound **5** (5-Br) showed the highest antifungal activity; with a 29-mm zone of inhibition against *Candida parapsilosis* RCMB 05065 and 26 mm against *Candida albicans* RCMB 05035, *Candida tropicalis* RCMB 05049 and *Aspergillus fumigatus* RCMB 02564 (Table 4). Seven compounds, **2**–**6**, **14** and **20**, from the series of 5-substituted benzimidazole derivatives also showed promising antifungal activity (Table 4).

Interestingly, all of the selected 23 compounds showed antifungal activity against both the unicellular and filamentous fungi (Table 4). Moreover, the minimum inhibitory concentration (MIC) of some compounds, against certain fungal species, was better (*i.e*., lower) than that of the reference drug (Table 5). This suggests that when there is no substituent at position 2 and an electron withdrawing substituent at position 5, the antifungal activity is increased (Table 4 and Table 5). This confirms the hypothesis that there is a direct relationship between the antifungal activity and the electron withdrawing effect of substituents. Compound **5** (5-Br) was active against the selected strains with MIC equivalent to half that exhibited by amphotericin B [31].

The antifungal screening of the series of 2-alkylamine benzimidazole derivatives (**9**–**19**, Scheme 1) showed interesting antifungal activity for the compounds. Significant activity was observed for Compounds **11** (5-Cl) and **14** (5-NO_2_), especially against *Mucor circinelloides* RCMB 07328 with low MICs of 3.9 and 0.49 µg/mL, respectively. However, no activity was observed for the 5-bromo analogue **12**. Both Compounds **11** and **14** showed remarkable antifungal activity that was almost similar to the reference drug, and interestingly, Compound **14** was three-fold as potent as the amphotericin B against *Mucor circinelloides* RCMB 07328. This suggests that the more electrons withdrawing the substituent there are, the higher the antifungal activity (Table 5). Furthermore, Compounds **11** (5-Cl), **13** (5-F), **14** (5-NO_2_) and **15** (5-H) showed good activity against *Candida parapsilosis* RCMB 05065; with a MIC range of 1.95–0.06 µg/mL, which is for Compounds **11** and **15** equivalent to the MIC of amphotericin B. Compound **13**, the 5-fluoro analogue, was three-times more potent, while **14** (5-NO_2_) was five-fold more potent than the control drug against *Candida parapsilosis* RCMB 05065. This suggests that when the electron withdrawing effect increases, the antifungal effect also increases.

Compound **27** (5-Br), from the series of 2-methanol benzimidazole derivatives (Scheme 2), exhibited promising antifungal activity against *Absidia corymbifera* RCMB 09635, *Syncephalastrum racemosum* RCMB 05922 and *Candida krusei* RCMB 05051 with MICs equivalent to the reference, and two-fold the latter’s potency against *Candida parapsilosis* RCMB 05065. This suggests that the more electronegative bromine increases the antifungal activity (Table 5) [29].

**Table 4:** Antifungal activity of the benzimidazole derivatives against unicellular and filamentous fungi in the well diffusion assay.

Fungal Strain	Diameter of Zone of Inhibition (mm) around Each Compound																							
	2	3	4	5	6	7	11	13	14	15	20	22	27	28	33	37	38	42	43	44	51	52	53	Am. B
**Unicellular**																								
*Candida albicans* RCMB 05035	21	21	**24**	**26**	**23**	21	21	20	**22**	19	**23**	17	20	18	19	17	**22**	17	19	20	19	21	**22**	**22**
*Candida krusei* RCMB 05051	17	16	**19**	**19**	18	14	14	12	17	14	**20**	17	**19**	0	0	0	18	0	0	0	0	**21**	**22**	**19**
*Candida parapsilosis* RCMB 05065	**21**	**21**	**25**	**29**	**23**	**19**	**19**	**22**	**24**	**19**	**20**	15	**21**	**19**	**21**	**18**	**22**	**18**	16	**20**	16	**18**	**18**	**18**
*Candida tropicalis* RCMB 05049	22	22	**25**	**26**	**25**	21	20	22	22	19	21	15	21	19	20	18	22	18	20	19	20	22	24	**25**
**Filamentous**																								
*Absidia corymbifera* RCMB 09635	**21**	**21**	**22**	**25**	**23**	15	14	19	**21**	14	0	0	19	19	19	17	**20**	16	18	18	**20**	0	0	**20**
*Aspergillus clavatus* RCMB 02593	**20**	21	**23**	**23**	**23**	17	19	19	21	17	**23**	19	18	14	15	15	20	14	16	15	18	**22**	**23**	**22**
*Aspergillus fumigatus* RCMB 02564	22	21	**24**	**26**	**23**	19	19	20	**23**	19	**23**	18	17	16	17	14	20	16	17	17	18	20	21	**24**
*Mucor circinelloides* RCMB 07328	**20**	**21**	**21**	**23**	**21**	17	**18**	17	**21**	17	**24**	**19**	15	17	**18**	14	**18**	12	14	14	**20**	**23**	**25**	**18**
*Penicillium marneffei* RCMB 01267	19	19	**22**	**25**	**23**	17	16	13	19	15	**21**	16	17	18	17	18	20	15	16	17	19	**21**	**22**	**21**
*Syncephalastrum racemosum* RCMB 05922	19	19	**20**	**20**	19	13	18	18	19	17	**24**	**21**	**20**	0	0	0	19	0	0	0	**0**	**23**	**24**	**20**

Good activity, relative to the diameter of the zone of inhibition obtained with amphotericin B (*i.e.*, a diameter ≥ amphotericin B (Am. B)), is indicated in bold.

**Table 5:** MICs of the tested compounds against unicellular and filamentous fungi.

Fungal Strain	MIC (µg/mL) per Compound																							
	2	3	4	5	6	7	11	13	14	15	20	22	27	28	33	37	38	42	43	44	51	52	53	Am. B
**Unicellular**																								
*Candida albicans* RCMB 05035	0.49	0.98	**0.03**	**0.007**	**0.03**	0.49	0.49	0.49	0.24	1.95	0.24	31.3	0.98	3.9	1.95	7.8	0.24	7.8	0.98	0.98	0.98	0.98	0.98	**0.12**
*Candida krusei* RCMB 05051	7.8	15.6	1.95	**0.98**	**0.98**	31.25	62.5	31.3	7.8	62.5	1.95	15.6	**0.98**	>500	>500	>500	1.95	>500	>500	>500	>500	**0.98**	**0.94**	**0.98**
*Candida parapsilosis* RCMB 05065	**0.24**	**0.12**	**0.03**	**0.003**	**0.06**	**0.98**	**1.95**	**0.24**	**0.06**	**1.95**	**1.95**	125	**0.49**	**1.95**	**0.24**	**1.95**	**0.12**	**1.95**	**0.98**	**0.98**	15.6	7.8	7.8	**1.95**
*Candida tropicalis* RCMB 05049	0.24	0.24	**0.02**	**0.007**	0.06	0.49	0.98	0.24	0.12	0.98	1.95	62.5	0.49	1.95	0.49	3.9	0.12	3.9	1.95	1.95	0.49	0.49	0.12	**0.02**
**Filamentous**																								
*Absidia corymbifera* RCMB 09635	**0.24**	**0.24**	**0.12**	**0.03**	**0.06**	15.63	31.3	1.95	**0.49**	62.5	>500	>500	**0.98**	3.9	1.95	7.8	0.98	7.8	7.8	3.9	**0.98**	>500	>500	**0.98**
*Aspergillus clavatus* RCMB 02593	0.49	0.98	**0.12**	**0.06**	**0.12**	3.9	1.95	1.95	0.24	3.9	0.24	3.9	1.95	62.5	31.3	31.3	0.98	62.5	15.6	31.3	3.9	0.49	0.24	**0.12**
*Aspergillus fumigatus* RCMB 02564	0.24	0.24	**0.06**	**0.02**	**0.06**	1.95	1.95	0.98	0.12	1.95	0.49	7.8	3.9	15.6	7.8	62.5	0.49	15.6	7.8	7.8	1.95	1.95	0.98	**0.06**
*Mucor circinelloides* RCMB 07328	**0.49**	**0.49**	**0.24**	**0.12**	**0.12**	7.8	**3.9**	7.8	**0.49**	15.6	**0.12**	**1.95**	31.3	7.8	1.95	62.5	1.95	125	62.5	62.5	**0.98**	**0.24**	**0.12**	**3.9**
*Penicillium marneffei* RCMB 01267	1.95	0.98	**0.12**	**0.03**	**0.03**	7.8	15.6	62.5	0.98	15.6	1.95	31.3	3.9	1.95	3.9	1.95	0.98	15.6	15.6	7.8	1.95	0.98	**0.49**	**0.49**
*Syncephalastrum racemosum* RCMB 05922	1.95	1.95	**0.98**	**0.98**	**0.98**	125	3.9	3.9	**0.98**	3.9	**0.24**	**0.98**	**0.98**	>500	>500	>500	1.95	>500	>500	>500	>500	**0.24**	**0.12**	**0.98**

Good activity, relative to the MIC of amphotericin B (Am. B), is indicated in **bold**. The results are the average of three independent readings.

In the 2-carboxylic acid benzimidazole series (Scheme 2), good antifungal activity was obtained for two compounds, **43** (5-Cl) and **44** (5-Br), with a MIC of 0.98 µg/mL only against *Candida parapsilosis* RCMB 05065, which is twice the potency exhibited by the reference drug. However, when comparing these results to their unsubstituted (at position 2) analogues from series of 5-substituted benzimidazoles (**5**, 5-Br), the latter compounds were more active antifungals. Therefore, this result confirmed that the activity is dependent on the halogen substituents at position 5 (Table 5) [29].

Antifungal activity for two compounds in the 2-chloromethyl benzimidazole series (Scheme 2), **33** (5-Me) and **38** (5-NO_2_), showed MICs of 1.95 µg/mL against *Mucor circinelloides* RCMB 07328, which is one-fold as potent as amphotericin B. In addition, the MICs for these compounds were higher than the standard drug by three- to four-fold against *Candida parapsilosis* RCMB 05065 (Table 5). This could be explained, in the case of **38** (5-NO_2_), which was more active than the methyl derivative **33**, by the presence of the highly electron withdrawing substituent. As a result, Compound **38** (5-NO_2_) is almost two times as active as Compound **33** (5-Me) (Table 5).

Compound **20** (5-H), from the 2-ethylbenzimidazole series (Scheme 1), exhibited good activity against *Mucor circinelloides* RCMB 07328 (MIC of 0.12 µg/mL; five-fold the potency of amphotericin B) and against *Syncephalastrum racemosum* RCMB 05922 (MIC of 0.24 µg/mL; three-fold the potency of amphotericin B) (Table 5). Interestingly, Compound **20**, which is unsubstituted at position 5, showed promising activity, while the nitro derivative **21** was inactive (Table 5). Surprisingly, the inhibition activity decreases when the electron withdrawing effect of the substituent at position 5 increases; this is in contrast to previous results [32].

## 3. Experimental Section

### 3.1. Chemistry: General Methods

The melting points (m.p.) were determined using a Gallenkamp melting point apparatus. The IR spectra were recorded in KBr discs on a Nicolet 140 FTIR spectrophotometer (ν_max_ in cm^−1^). ^1^H- and ^13^C-NMR spectra were recorded using a Bruker DPX400 spectrometer. Coupling constants are given in Hertz (Hz). Deuterated solvents were obtained from Goss Scientific Instruments. Deuterated chloroform was stored over 4 Å molecular sieves. DMSO-*d*_6_ and D_2_O were stored in silica gel desiccators. The mass spectra were obtained on electron impact using an AEI MS902 mass spectrometer. Elemental analysis was carried out by the microanalysis service using 2 mg of the sample (MEDAC LTD, Analytical and Chemical Consultancy Services, Surrey, UK). Spectral data (IR, NMR and mass spectra) confirmed the structures of the compounds.

The purity of all of the compounds was established by ^1^H- and ^13^C-NMR spectroscopy. All of the known compounds were identified by comparing their analytical and physicochemical data with previously reported data. The data for the compounds is provided in the Supplementary Information.

### 3.2. Screening for Antibacterial Activity

#### 3.2.1. Bacterial Strains Used in This Project

All reference and clinical strains were obtained from Dr. Anna Snelling, University of Bradford. Overall, 15 strains of *Staphylococcus* spp. (including 2 × *S. aureus*, 10 × MRSA, 2 × *S. epidermidis* and 1 × *S. haemolyticus*), 5 strains of *E. coli*, 4 strains of *Ps. aeruginosa*, 1 strain of *Serratia marcescens* and 1 strain of *Burkholderia cepacia* were used.

#### 3.2.2. Screening for Antibacterial Activity by the Disc Diffusion Method

Disc diffusion assays for antimicrobial susceptibility testing were carried out in-line with the standard method first described by Bauer (1966), to assess if the compounds had any appreciable antibacterial activities. The broad spectrum antibiotic ciprofloxacin was included in these qualitative tests as a comparator and positive control. Bacterial suspensions were prepared from fresh, overnight, agar cultures in sterile distilled water and adjusted to a 0.5 McFarland turbidity standard (Remel, Lenexa, KS, USA). These suspensions were used to seed Iso-sensitest agar (Oxoid, Hampshire, UK) plates evenly using a sterile swab [33].

For test compounds, each was dissolved to 10 mg/mL in DMSO, and different amounts of each (typically 10, 100 and 200 µg) were loaded from the working stock solution onto 6-mm diameter sterile Whatmann paper discs (Fisher Scientific, Loughborough, UK) and allowed to dry. The loaded discs were placed flat on the surface of the agar lawns. Each test plate was comprised of five discs: three impregnated, one positive control (ciprofloxacin 5 µg) and one negative control (impregnated with 20 µL of 100% DMSO). The plates were incubated at 37 °C for 24 h. At the end of incubation, the plates were examined for zones of inhibition. Diameters of inhibition zones were measured (in mm) and recorded. If any activity was observed, the test was repeated on a separate day, using another fresh culture.

#### 3.2.3. Determination of Minimum Inhibitory Concentration of Compounds against Bacterial Strains

For quantitative assessment of activity, the MICs of the synthesised compounds plus ciprofloxacin were determined by the agar dilution method using Iso-sensitest agar. Agar dilution was in the range of 0.5–512 µg/mL of each compound, in doubling dilutions. Iso-sensitest agar was sterilised in 19-mL aliquots, using sufficient base powder for 20 mL. Once it had cooled to approximately 50 °C, an appropriate amount of the test compound was added in a volume of 1 mL, to give the desired final concentration per plate. Bacterial suspensions were prepared as in the previous section.

Strains were seeded (~10 µL) on to the freshly-prepared MIC agar plates using a multi-point inoculator (Denley, Guangzhou, China). After briefly allowing the inocula to dry, the plates were incubated overnight at 37 °C and the MIC was recorded as the lowest concentration of compound that inhibited bacterial growth completely, as indicated by the absence of any visible growth in the inoculation spot on the agar surface.

### 3.3. Screening for Antifungal Activity

#### 3.3.1. Fungal Strains Used in This Project

The strains were obtained from the Regional Center for Mycology and Biotechnology (RCMB), Al-Azhar University, Cairo, Egypt, and this part of the practical work was done there, as well. Reference isolates for the antifungal screening; included representatives of unicellular fungi, namely *Candida albicans* RCMB 05035, *Candida krusei* RCMB 05051, *Candida parapsilosis* RCMB 05065 and *Candida tropicalis* RCMB 05049, and filamentous fungi, namely *Absidia corymbifera* RCMB 09635, *Aspergillus clavatus* RCMB 2593, *Aspergillus fumigatus* RCMB 02564, *Mucor circinelloides* RCMB 07328, *Mucor circinelloides* RCMB 07328, *Absidia corymbifera* RCMB 09635, *Penicillium marneffei* RCMB 01267 and *Syncephalastrum racemosum* RCMB 05922.

#### 3.3.2. Screening for Antifungal Activity by the Well Diffusion Method

A qualitative assessment of antifungal activity was determined by the well diffusion method according to the National Committee for Clinical Laboratory Standards (NCCLS) methodology (National Committee for Clinical Laboratory Standards, 1993). Petri dishes containing 20 mL of malt extract agar (Oxoid) were seeded with 2–3 day old cultures of fungal inoculums (suspensions of spores in sterile water). Wells (6 mm in diameter) were cut into the agar with a sterile cork borer, and 50 μL of compound diluted in DMSO were added at a concentration of 5 mg/mL (250 μg per well). Plates were incubated at 37 °C (unicellular strains) or 28 °C (filamentous strains) for 3–7 days depending on the growth rate of each strain. Antifungal activity was determined based on the measurement of the diameter of the inhibition zone formed around each well.

#### 3.3.3. Determination of Minimum Inhibitory Concentration of Compounds against Fungal Strains

For quantitative assessment of activity, chemical compounds showing activity in the well diffusion tests were diluted in DMSO, and then serial doubling dilutions were made in sterile growth media. The commonly-used antifungal drug amphotericin B (Sigma-Aldrich A9528, St. Louis, MO, USA) was included as a comparator. Dilutions were placed in separate wells of a 96-well microtitre tray (Sarstedt). A volume of standardized inoculum equal to the volume of the diluted compound was added to each well, bringing the microbial concentration to approximately 5 × 10^5^ cells per mL. The trays were incubated at a temperature appropriate for the test species (28 °C or 37 °C), for a pre-set period (2–3 days). After incubation, the series of dilution wells was observed for fungal growth, usually indicated by turbidity and/or a pellet of fungi in the bottom of the well. For each test compound, the lowest concentration that completely inhibited growth was recorded as its minimum inhibitory concentration (MIC).

## 4. Conclusions

**Figure 2:**
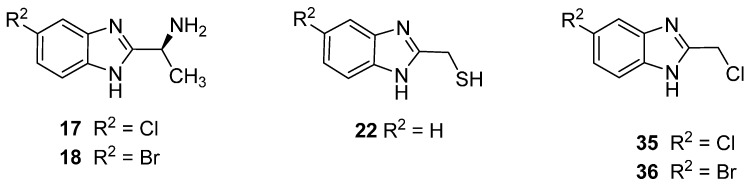
Promising antibacterial derivatives.

**Figure 3:**
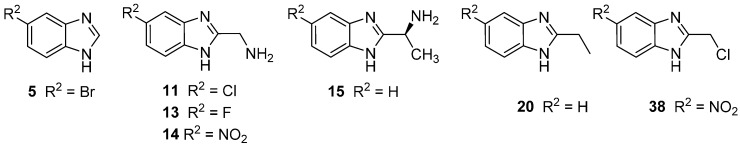
Promising antifungal derivatives.

A series 53 compounds were synthesized based on the 2-benzimidazole nucleus. While none of the compounds were novel, 47 of them were herein tested against a panel of strains of bacteria and fungi for the first time. Infections caused by MRSA variants of *S. aureus* are particularly difficult to treat, as the strains are resistant to β-lactams and often manifest resistance to other classes of drug, too, such as the fluoroquinolones. Thus, compounds active against the MRSA strains used in this study can be considered promising lead compounds. In terms of the SAR, the key factor in this case is the presence of the chlorine or bromine substituent at position 5 of the benzimidazole ring, and the presence of CH(CH_3_)NH_2_ or CH_2_Cl at position 2. Moreover, the presence of CH_2_SH at position 2 (H at position 5) proved effective for antimicrobial activity. Derivatives with CH_2_NH_2_ were not active against bacteria, but when a branched methyl group is added (CH(CH_3_)NH_2_), the antibacterial activity is improved. The latter compounds were prepared from *S*-alanine. Therefore, the product, which is also chiral, showed variable activity against the MRSA species. Chirality is a feature for many drugs, with one of the stereoisomers active biologically and the other inactive or even toxic. Therefore, the chirality has a significant impact on enhancing the biological activity of the compound. However, as in the case of the antifungal assay, and according to the SAR study, an extremely important factor is the nature of the substituent attached to the aromatic ring. The presence of the halogen atom (Cl, Br, F) at position 5, increased the antifungal activity. For the 5-nitro derivative, the presence of CH_2_NH_2_ at position 2 increased the activity. The *N*-methylated-2-methanolbenzimidazole derivatives were more active than the unmethylated analogues, and this is observed when the substituent at position 5 was F, OMe or NO_2_. To summarise, according to the SAR analysis, it can be concluded that some derivatives of benzimidazole offer significant possibilities for the development of new broad-spectrum antimicrobial agents. Through appropriate modification and fine-tuning of substituents at positions 1, 2 and 5, new microbial inhibitors are possible, although thorough toxicological evaluation will be needed to assess their utility for medicinal use. To summarise, according to the SAR analysis, it can be concluded that some derivatives of benzimidazole offer significant possibilities for the development of new broad-spectrum antimicrobial agents. The structures of our lead compounds are shown in (Figure 2 and Figure 3).

## Supplementary Materials

Supplementary materials can be accessed at: http://www.mdpi.com/1420-3049/20/08/15206/s1.
