# Supplementary material for: Synthesis and Evaluation of Selected Benzimidazole Derivatives as Potential Antimicrobial Agents
Source: Molecules. 2015 Aug 20;20(8):15206–23. doi: 10.3390/molecules200815206 (PMC6332381; doi:10.3390/molecules200815206)
Supplement: Supplementary file 1 [file molecules-20-15206-s001.pdf]

Supplementary Data

Table S1. Reaction time, yields and melting points of the synthesized benzimidazole derivatives.

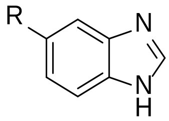

Compound	R	Reaction Time (min)	Yield (%)	mp/°C	Lit. mp/°C
**1**	H	30	74	170–172	170 [1]
**2**	CH_3	60	87	112–114	112–114 [2]
**3**	OCH_3	60	85	116–118	117–120 [3]
**4**	Cl	180	100	123–125	124–126 [4]
**5**	Br	180	97.5	130–133	130–131 [5]
**6**	F	180	90.0	130–132	132 [6]
**7**	NO_2	480	100	203–205	204–205 [4]
**8**	CN	1440	44	230–232	230–233 [7]

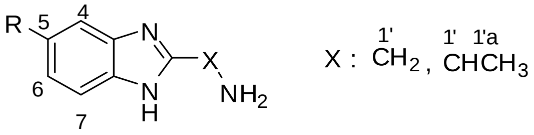

Compound	R	Amino Acid	Reaction Time (h)	Yield (%)	mp/°C	Lit. mp/°C
**9**	H	Gly	300	89	270–272	268–270 [8]
**10**	CH_3	Gly	170	52	208–210	208–210 [9]
**11**	Cl	Gly	216	39	252–254	251–252 [9]
**12**	Br	Gly	216	22	258–260	NEW
**13**	F	Gly	216	16	262–264	258–262 [9]
**14**	NO_2	Gly	300	21	254–256	248–251 [9]
**15**	H	*L*-Ala	264	67	138–140	133–138 [8]
**16**	CH_3	*L*-Ala	264	68.5	212–214	212–213 [9]
**17**	Cl	*L*-Ala	288	41	154–156	152–154 [9]
**18**	Br	*L*-Ala	288	19	158–160	NEW
**19**	F	*L*-Ala	288	33	260–262	262–264 [9]

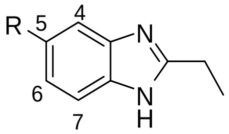

Compound	R	Reaction Time (h)	Yield (%)	mp/°C	Lit. mp/°C
**20**	H	24	60	164–166	164–165 [10]
**21**	NO_2	24	40	175–177	178–179 [11]

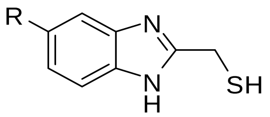

Compound	R	Reaction Time (h)	Yield (%)	mp/°C	Lit. mp/°C
**22**	H	60	82	170–172	173 [12]
**23**	NO_2	70	47.8	195–197	195 [12]

Table 1. *Cont.*

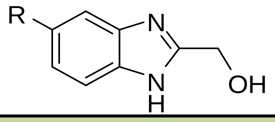

Compound	R	Reaction Time (min)	Yield (%)	mp/°C	Lit. mp/°C
**24**	H	180	96	172–174	170–172 [13]
**25**	CH_3	180	100	194–196	202–203 [9]
**26**	OCH_3	480	100	182–184	190–191 [14]
**27**	Cl	480	95	200–202	202 [13]
**28**	Br	480	85	208–210	208 [15]
**29**	F	480	70	182–184	182 [16]
**30**	NO_2	2880	56	198–200	198–200 [9]
**31**	CN	60	57	170–172	172–173 [17]

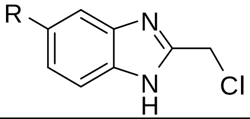

Compound	R	Reaction Time (min)	Yield (%)	Method	mp/°C	Lit. mp/°C
**32**	H	180	72	Hiroyuki, 2001	142–144	140–141
**33**	CH_3	180	61	Hiroyuki, 2001	128–-130	127–132
**34**	OCH3	180-	89	Dirk, 2009	200–202	199 ± 23.2 °C Calculated (ACD/LABS)
**35**	Cl	300-	79	Dirk, 2009	210–212	213–214 [18]
**36**	Br	300-	91	Dirk, 2009	248–250	249–250 [19]
**37**	F	300-	80	Dirk, 2009	212–214	180.6 ± 22.3 °C Calculated (ACD/LABS)
**38**	NO_2	480-	88	Dirk, 2009	170–172	174 [20]
**39**	CN	560-	57	Dirk, 2009	230–232	230–232 [7]

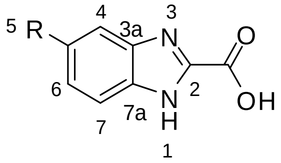

Compound	R	Yield (%)	mp/°C	Lit. mp/°C
**40**	H	44	170–172	(lit. 168–169 °C) [21]
**41**	CH3	83	140–142	(lit., 140 °C) [22].
**42**	OCH3	84	150–152	(lit., 152–154 °C) [23]
**43**	Cl	24	158–160	(lit., 159–160 °C) [23].
**44**	Br	37	162–164	(lit., 206 °C) [24]
**45**	F	38	166–168	224.3 ± 26.5 °C Calculated (ACD/LABS)
**46**	NO2	55	198–200	(lit., 198–200 °C) [9].

Table 1. *Cont.*

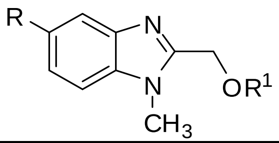

Compound	R	R[1]	Reaction Time (h)	Yield (%)	mp/°C	Lit. mp/ °C
**47**	H	H	24	43.4	128–130	125–130 [25]
**48**	CH_3	H	24	46.5	122–124	150 °C [26]
**49**	Cl	H	24	8.8	136–138	178.2 ± 22.3 °C Calculated (ACD/LABS)
**50**	Br	H	24	15.8	158–160	NEW
**51**	F	H	24	53	132–134	NEW
**52**	OCH_3	H	24	25	182–184	183 [27]
**53**	NO_2	H	24	19.4	160–162	167–168 [27]

New Compounds

(5-Bromo-1H-benzimidazole-2-yl)methanamine **12**

(5-Bromo-*1H*-benzimidazole-2-yl)methanamine **12** was prepared using the Phillips procedure [1], using 4-bromo-1,2-phenylenediamine (3.74 g, 20 mmol) and glycine (2.25 g, 30 mmol) dissolved in hydrochloric acid (14 mL, 5.5 M). The reaction was heated under reflux for 10 days and then left to evaporate slowly. 5-Bromo-(*1H*-benzimidazole-2-yl)methanamine was isolated as brownish orange precipitate Yield 22%. mp 258–260 °C. ^{1}H-NMR (DMSO-d_6) δ 9.093 (s, br, 2H, NH_2), δ 8.815 (br, s, 1H, N*H*), δ 7.36 (d,1H, ^4J = 4.8 Hz, 4-H), δ 8.11 (d,1H, ^3J = 8.8 Hz, 7-H), δ 7.77 (dd,1H, ^3J = 9.20 Hz, ^4J = 1.60 Hz, 6-H), δ 4.38 (s, 2H, 1′-H);^{13}C-NMR (DMSO-d_6) δ 168.98 (C-2), δ 149.58 (C-3a, C-7a), δ 129.41 (C-6), δ 126.55 (C-4), δ 122.51 (C-7), δ 106.86 (C-5), δ 36.30 (C-1′); ν_{max}/cm^{-1} (KBr) 3233 (N-H), 3056 (C-H, *sp*^{2}), 2852 (C-H, *sp*^{3}), 1074 C-Br. MS (EI): *m/z* 226 (M$^+$, 100%) 228 (M + 2, 100%).

(S)-1-(5-Bromo-1H-benzimidazole-2-yl)ethanamine **18**

(*S*)-1-(5-Bromo-*1H*-benzimidazole-2-yl)ethanamine **18** was prepared according to the Phillips procedure [1] using 4-bromo-1,2-phenylenediamine (0.22 g, 1.2 mmol), *S*-alanine (0.11 g, 1.23 mmol) and hydrochloric acid (20 mL, 5.5 M). The mixture was heated under reflux for 12 days. The resulting dark brown solution was evaporated to dryness. (*S*)-1-(5-bromo-*1H*-benzimidazole-2-yl)ethanamine was obtained as a dark brown solid Yield 19%. mp 158–160 °C. ^{1}H-NMR (DMSO-d_6) δ 8.13 (1H, t, ^3J = 9.20 Hz, 7-H), δ 7.78 (1H, d, ^3J = 9.20 Hz, 6-H), δ 7.33 (1H, d, ^4J = 4.40 Hz, 4-H), δ 4.73 (1H, m, 1′-H), δ 1.39 (3H, d, ^3J = 6.80 Hz, 2′). ν_{max}/cm^{-1} (KBr) 3401 (N-H), 3044 (C-H, *sp*^{2}), 2847 (C-H, *sp*^{3}), 1621 and 1471 (C=N). MS (EI): *m/z* 240 (M$^+$, 100%) 242 (M + 2, 100%).

(N-Methyl-5-bromo-1H-benzimidazole-2-yl)-methanol **50**

N-Methyl-2-methanol 5-bromobenzimidazole **50** was prepared according to the procedure by González-Chávez *et al.* [28]. A solution of 5-bromo-(*1H*-benzimidazole-2-yl)-methanol **28** (0.60 g, 2.60 mmol), and sodium hydroxide (0.10 g, 2.60 mmol) were stirred in dry acetone (10 mL) for 30 min.

Then, iodomethane (0.37 g, 2.60 mmol) was added and the mixture was stirred for 24 h. The reaction mixture was concentrated to a quarter and then poured into ice-cold water. The solid was filtered and washed with 50% HCl. The solid was washed with water (100 mL) and purified by column chromatography (9:1 chloroform/ethanol) to give the desired product (50) as a bright yellow crystals Yield 16%. mp 158–160 °C. ^{1}H-NMR (DMSO-d_6) δ 7.88 (1H, s, 4-H), δ 7.58 (1H, d, ^3J = 8.00 Hz, 7-H), δ 7.43 (1H, dd, ^3J = 8.00 Hz, ^4J = 1.60 Hz 6-H), δ 5.68 (1H, br, s, OH), δ 4.75 (2H, s, CH_2), δ 3.86 (3H, s, N-CH_3); ^{13}C-NMR (DMSO-d_6) δ 155.42 (C-2), δ 143.10 (C-3a), δ 137.27 (C-7a), δ 124.74 (C-5), δ 121.66 (C6), δ 120.55 (C-7), δ 111.90 (C-4) δ 56.27 (C-1′) δ 30.03 (N-CH_3); ν_{max}/cm^{-1} (KBr) 3122 (C-H, *sp*^{2}), 2851 (C-H, *sp*^{3}), 1479 and 1348 (C-H, bend), 1105 (C-Br); MS (EI): *m/z* 241 (M^+, 100%), 243 (M + 2, 100%).

(N-Methyl-5-fluoro-1H-benzimidazole-2-yl)-methanol **51**

N-Methyl-2-methanol-5-flourobenzimidazole **51** was prepared according to using the procedure by Harisha *et al*. [28]. A solution of (5-fluoro-*1H*-benzimidazole-2-yl) methanol **29** (0.42 g, 2.5 mmol), and sodium hydroxide (0.1 g, 2.5 mmol) were stirred in dry acetone (8 mL) for 30 min. Then, iodomethane (0.35 g, 2.5 mmol) was added and the mixture was stirred for 24 h. The reaction mixture was concentrated to a quarter and then poured into ice-cold water. The solid was filtered and washed with 50% HCl to neutralize the excess potassium carbonate. The solid was washed with cold water (25 mL) and aqueous ethanol. The desired product **51** was dried and recrystallized from ethanol to give pale cream crystals Yield 53%. mp 132–134 °C. ^{1}H-NMR (DMSO-d_6) δ 7.60 (1H, d, ^3J = 8.00 Hz, 7-H), δ 7.53 (1H, d, ^3J = 8.00 Hz, 4-H), δ 7.25 (1H, dt, ^3J = 7.80 Hz, ^4J = 1.20, 5-H), δ 7.19 (1H, dt, ^3J = 8.00 Hz, ^4J = 1.20, 6-H), δ 5.63 (1H, t, ^3J = 5.60 Hz, O*H*), δ 4.73 (2H, d, ^3J = 4.80 Hz, CH_2), δ 3.83 (3H, s, CH_3); ^{13}C-NMR (DMSO-d_6) δ 155.02 (C-4, C-7), δ 121.27 (C-5, C-6), δ 118.14 (C-3a, C-7a), δ 111.12 (C-2), δ 57.51 (C-1′); ν_{max}/cm^{-1} (KBr) 3170 (C-H, *sp*^{2}), 2865 (C-H, *sp*^{3}), 1434 and 1343 (C-H, bend), 1137 (C-F). MS (EI): *m/z* 181 (M + 1, 100%), 182 (M + 2, 15%), 163 (M-OH, 15%).

Other Compounds

((5-Methyl-1H-benzimidazole-2-yl)-methanol) **25**

(5-Methyl-1*H*-benzimidazole-2-yl)-methanol **25** was prepared using the Phillips procedure [1], 4-Methyl-1,2-phenylenediamine (12.22 g; 0.1 mol) and glycolic acid (11.40 g; 0.15 mmol) in hydrochloric acid (50 ml, 5.5 M) were heated under reflux with for 3 h. The reaction mixture was cooled to room temperature and ammonia solution was added and the mixture cooled in ice until a bright brown precipitate formed. The resulting solid was recrystallised from aqueous ethanol to give (5-methyl-1*H*-benzimidazole-2-yl)-methanol as a pale creamy powdery solid Yield 100%. mp 194–196 °C (lit. 203 °C) [29]. ^{1}H-NMR (DMSO-d_6) δ 7.38 (1H, d, ^3J = 8.40 Hz, 7-H), δ 7.29 (1H, s, 4-H), δ 6.97 (1H, d, ^3J = 8.40 Hz, 6-H), δ 4.69 (2H, s, CH_2), δ 2.39 (3H, s, CH_3), NH not observed; ^{13}C-NMR (DMSO-d_6) δ 154.73 (C-2), δ 137.96 (C-3a), δ 136.6 (C-7a), δ 130.4 (C-6), δ 122.9 (C-5), δ 114.64 (C-4), δ 114.13 (C-7), δ 57.52 (C-1′), δ 21.20 ($\underline{C}H_3$); ν_{max}/cm^{-1} (KBr) 3481 (O-H), 3314 (NH) 3050 (C-H, *sp*^{2}), 2850 (C-H, *sp*^{3}).

((5-Methyl-1H-benzimidazole-2-yl)-methanol) **26**

(5-Methoxy-1*H*-benzimidazole-2-yl)-methanol **26** was prepared using the Phillips procedure [1], 4-Methoxy-1,2-phenylenediamine (0.7 g, 5 mmol) was heated under reflux temperature with glycolic acid (0.4 g, 5.25 mmol) in hydrochloric acid (15 mL, 5.5 M) for 6 h. The reaction mixture was cooled to room temperature and ammonia solution was added and the mixture cooled in ice until a bright brown precipitate formed. The resulting solid was recrystallised from aqueous ethanol to give 5-methoxy-*1H*-benzimidazole-2-yl)-methanol as a bright brown solid Yield 100%. mp 182–184 °C. ^{1}H-NMR (DMSO-d_6) δ 7.37 (1H, d, ^{3}J = 8.72 Hz, 7-H), δ 6.99 (1H, d, ^{4}J = 2.36 Hz, 4-H), δ 6.76 (1H, dd, ^{3}J = 8.64 Hz, ^{4}J = 2.48 Hz, 6-H), δ 4.75 (2H, s, C*H*2), 3.75 (3H, s, OCH_3), 5.65 (1H, br, s, OH), δ 12.20 (1H, br, s, NH). ^{13}C-NMR (DMSO-d_6) δ 115.74 (C-2), δ 155.20 (C-3a,C-7a), δ 97.27 (C-5), δ 110.56 (C-4, C-6), δ 154.50 (C-7), δ 57.68 (C-1′), δ 55.35 (O<u>C</u>H3); MS (EI): *m*/*z* 177 (M-1, 100%), 162 (M-OH, 10%). ν_{max}/cm^{-1} (KBr) 3392 (O-H), 3110 (C-H, *sp*^{2}), 3038 (C-H, *sp*^{3}), 1459 and 1378 (C-H, bend); MS (EI): *m*/*z* 178 (M$^+$, 100%). Found; C, 60.47%; H, 5.30%; N, 15.92%, requires; C, 60.66%; H, 5.66%; N, 15.72%.

2-(Chloromethyl)-5-methoxy-1H-benzimidazole hydrochloride salt **34**

Thionyl chloride (27.5 mmol, 2 mL) was added slowly to a solution of 5-methoxy-*1H*-benzimidazole-2-yl)-methanol) **26** (0.58 g, 3.31 mmol) in dichloromethane (10 mL) at 10 °C, the mixture was stirred until no presence of the starting material. The solvent was then evaporated, and the residue was triturated with DCM, and suction filtered, then was washed with dichloromethane and ether. Yield 89% which **34** was recovered as a green powder. mp 200–202 °C. ^{1}H-NMR (DMSO-d_6) δ 7.72 (1H, d, ^{3}J = 9.20 Hz, 7-H), δ 7.25 (1H, d, ^{4}J = 2.00 Hz, 4-H), δ 7.16 (1H, d, ^{3}J = 9.00 Hz, ^{4}J = 2.40 Hz, 6-H), δ 5.22 (2H, s, CH_2), δ 3.85 (3H, s, OCH_3). ^{13}C-NMR (DMSO-d_6) δ 157.44 (C-2), δ 146.77 (C-3a), δ 131.50 (C-7a), δ 124.76 (C-6), δ 115.64 (C-5), δ 114.54 (C-4), δ 95.47 (C-7), δ 35.15 (C-1′), δ 33.26 (O<u>C</u>H3). ν_{max}/cm^{-1} (KBr) 3401 (NH) 3093 (C-H, *sp*^{2}), 2850 (C-H, *sp*^{3}), 1272 (<u>C</u>H2-Cl). MS (EI): *m*/*z* 197 (M$^+$, 100%), 199 (M + 2, 40%). Found; C, 46.09%; H, 4.08%; N, 11.83%, requires; C, 46.37%; H, 4.32%; N, 12.02%.

2-(Chloromethyl)-5-flouro-1H-benzimidazole **37**

Thionyl chloride (27.5 mmol, 2 mL) was added slowly to a solution of 5-flouro-1*H*-benzimidazole-2-yl)-methanol) (FAS27) (0.55 g, 3.31 mmol) in dichloromethane (10 mL) at 10 °C, the mixture was stirred until no presence of the starting material. The solvent was then evaporated, and the residue was triturated with dichloromethane, suction filtered, washed with dichloromethane and ether. 2-(Chloromethyl)-5-flouro-*1H*-benzimidazole **45** was recovered as a brown powder in Yield 80%. mp 212–214 °C. ^{1}H-NMR (DMSO-d_6) δ 7.82 (1H, d, ^{3}J = 8.80 Hz, 7-H), δ 7.67 (1H, d, ^{4}J = 2.00 Hz, 4-H), δ 7.38 (1H, d, ^{3}J = 8.80 Hz, ^{4}J = 2.00 Hz, 6-H), δ 5.18 (2H, s, CH_2) (lit., 4.87 (2H, s, CH_2), δ 7.05 (1H, td, ^{3}J = 9.00 Hz, ^{4}J = 3.00 Hz) δ 7.27 (1H, dd, ^{3}J = 9.00 Hz, ^{4}J = 3.00 Hz), δ 7.51–7.55 (1H, m)) [30]; ^{13}C-NMR (DMSO-d_6) δ 158.63 (C-2), δ 149.99 (C-3a), δ 132.97 (C-7a), δ 129.33.14 (C-6), δ 116.14 (C-5), δ 114.17 (C-4), δ 101.11 (C-7), δ 35.00 (C-1′); ν_{max}/cm^{-1} (KBr) 3434 (NH) 3083 (C-H, *sp*^{2}), 2797 (C-H, *sp*^{3}), 1219 (<u>C</u>H2-Cl); MS (EI): *m*/*z* 185 (M$^+$, 100%), 187 (M + 2, 30%).

5-Bromo-1H-benzimidazole-2-carboxylic Acid **44**

Synthesis of 5-bromo-*1H*-benzimidazole-2-carboxylic acid hydrochloride 44 was prepared using 5-bromo-*1H*-benzimidazole-2-yl)-methanol **28** (0.23 g, 1.0 mmol) dissolved in acetone (10 mL) and potassium permanganate (0379, 2.4 mmol) dissolve in water (10 mL). The crude product was recrystallised from water. 5-Bromo-*1H*-benzimidazole-2-carboxylic acid in Yield 37% was recovered as a creamy white powder. mp 162–164 °C. ^{1}H-NMR (DMSO-d_6) δ 10.80 (1H, s, N*H*), δ 8.44 (1H, s, O*H*), δ 7.84 (1H, d, ^{4}J = 3.20 Hz, 4-H), δ 7.62 (1H, d, ^{3}J = 8.80 Hz, 7-H), δ 7.47 (1H, dd, ^{3}J = 6.80 Hz, ^{4}J = 5.40 Hz, 6-H). ^{13}C-NMR (DMSO-d_6) δ 160.1 (C=O), δ 144.30 (C-2), δ 139.80 (C-3a), δ 137.80 (C-7a), δ 127.30 (C-5), δ 124.80 (C-6), δ 119.70 (C-7), δ 116.30 (C-4); ν_{max}/cm^{-1} (KBr) 3062 (C-H, *sp*^{2}), 1744 (C=O), 1046, (C-Br); (lit., ESI + 241) [31].

5-Fluoro-1H-benzimidazole-2-carboxylic acid **45**

5-Fluoro-*1H*-benzimidazole-2-carboxylic acid **45** was synthesised using 5-fluoro-*1H*-benzimidazole-2-yl)methanol **29** (0.10 g, 0.60 mmol) dissolved in acetone (20 mL) and potassium permanganate (0.10 g, 0.63 mmol) dissolved in water (10 mL). 5-Fluoro-*1H*-benzimidazole-2-carboxylic acid **45** was recovered as bright white crystals in Yield 38%. mp 166–168 °C. ^{1}H-NMR (DMSO-d_6) δ 12.14 (1H, br, s, O*H*), δ 7.80 (1H, ^{3}J = 8.00 Hz, ^{4}J = 3.20 Hz, 7-H), δ 7.53 (1H, d, ^{3}J = 8.00 Hz, 4-H), δ 7.32 (1H, t, ^{3}J = 8.00 Hz, 6-H); ^{13}C-NMR (DMSO-d_6) δ 165.43 (C=O), δ 159.68 (C-5), δ 142.78 (C-2), δ 141.80 (C-3a), δ 132.30 (C-7a), δ 112.02 (C-7), δ 113.90 (C-6), δ 104.20 (C-4); ν_{max}/cm^{-1} (KBr) 3433 (O-H), 3108 (C-H, *sp*^{2}), 1748 (C=O), 1335 (C-O), 1199 (C-F) ; (lit., ESI + 181) [31].

(N-Methyl-5-methyl-1H-benzimidazole-2-yl)-methanol **48**

N-Methyl-2-methanol-5-methylbenzimidazole **48** was prepared according to using the procedure by Harisha *et al.* [28]. A solution of 5-methyl-(*1H*-benzimidazole-2-yl)-methanol **25** (1.62 g, 0.01 mol), and sodium hydroxide (0.40 g, 0.01 mol) were stirred in dry acetone (30 mL) was stirred for 30 min. Then, iodomethane (1.41 g, 0.01 mol) was added to the mixture and the mixture was stirred for 24 h. The reaction mixture was concentrated to a quarter and then poured into ice-cold water. The solid was filtered and washed with 50% HCl to neutralize the excess potassium carbonate. Then, wash the solid with cold water (100 mL) and then aqueous ethanol. The product was purified by column chromatography (9:1 chloroform/ethanol) to give the desired product (FAS37) as a brown powder Yield 46%. mp 122–124 °C. ^{1}H-NMR (DMSO-d_6) δ 7.46 (1H, d, ^{3}J = 8.00 Hz, 7-H), δ 7.31 (1H, s, 4-H), δ 7.39 (1H, dd, ^{3}J = 9.80 Hz, ^{4}J = 2.80 Hz, 6-H), δ 5.59 (1H, s, O*H*,), δ 4.67 (2H, s, CH_2), δ 3.79 (3H, s, N-CH_3), δ 2.40 (3H, s, CH_3); ^{13}C-NMR (DMSO-d_6) δ 153.8 (C-2), δ 142.06 (C-3a), δ 136.24 (C-7a), δ 131.34 (C-6), δ 123.44 (C-5), δ 118.63 (C-4), δ 109.64 (C-7), δ 55.40 (C-1′), δ 21.39 ($\underline{C}H_3$), δ 29.76 ($\underline{C}H_3$); ν_{max}/cm^{-1} (KBr) 3154 (C-H, *sp*^{2}), 2853 (C-H, *sp*^{3}). MS (EI): *m*/*z* 176 (M^+, 100%).

(N-Methyl-5-chloro-1H-benzimidazole-2-yl)-methanol **49**

N-Methyl-2-methanol-5-chlorobenzimidazole **49** was prepared according to using the procedure by Harisha *et al.* [28] A solution of 5-chloro-(*1H*-benzimidazole-2-yl)-methanol **28** (1.50 g, 8.20 mmol), and sodium hydroxide (0.33 g, 8.2 mmol) were stirred in dry acetone (30 mL) for 30 min. Then,

iodomethane (1.41 g, 8.20 mmol) was added and the mixture was stirred for 24 h. The reaction mixture was concentrated to a quarter and then poured into ice-cold water. The solid was filtered and washed with 50% HCl to neutralize the excess potassium carbonate. The solid was washed with cold water (100 mL) and aqueous ethanol. The product was purified by column chromatography (9:1 chloroform/ethanol) to give the desired product **38** as bright orange crystals Yield 9%. mp 136–138 °C. ^{1}H-NMR (DMSO-d_6) δ 7.64 (1H, s, 4-H), δ 7.58 (1H, d, ^{3}J = 8.00 Hz, ^{4}J = 2.80 Hz, 7-H), δ 7.19 (1H, d, ^{3}J = 8.00 Hz, 6-H), δ 5.64 (1H, br, s, OH), δ 4.71 (2H, s, CH_2), δ 3.82 (3H, s, N-CH_3); ^{13}C-NMR (DMSO-d_6) δ 155.57 (C-2), 142.51 (C-3a), δ 136.78 (C-7a), δ 126.66 (C-5), δ 122.19(C6), δ 120.09 (C-7), δ 111.40 (C-4) δ 56.31 (C-1′) δ 30.03 (N-CH_3); ν_{max}/cm^{-1} (KBr) 3154 (C-H, *sp*^{2}), 2853 (C-H, *sp*^{3}), 1439 and 1334 (C-H, bend), 1134 (C-Cl). MS (EI): *m*/*z* 197 (M^+, 100%), 199 (M + 2, 40%). Found; C, 54.65%; H, 4.64%; N, 14.12%, requires; C, 54.97%; H, 4.61%; N, 14.25%.

(N-Methyl-5-nitro-1H-benzimidazole-2-yl)-methanol **53**

N-Methyl-2-methanol-5-nitrobenzimidazole **53** was prepared according to using the procedure by Harisha *et al*. [28]. A solution of 5-nitro-(*1H*-benzimidazole-2-yl)-methanol **30** (0.86 g, 4.50 mmol), and sodium hydroxide (0.18 g, 4.50 mmol) were stirred in dry acetone (20 mL) for 30 min. Then, iodomethane (0.64 g, 4.50 mmol) was added and the mixture was stirred for 24 h. The reaction mixture was concentrated to a quarter and then poured into ice-cold water. The solid was filtered and washed with 50% HCl to neutralize the excess potassium carbonate. The solid was washed with cold water (100 mL) and aqueous ethanol. The product was purified by column chromatography (9:1 chloroform/ethanol) to give the desired product (FAS50) as an yellow crystals Yield 19%. mp (160–162 °C) (lit., 167–168 °C [27]. ^{1}H-NMR (DMSO-d_6) δ 7.47 (1H, d, ^{4}J = 1.60 Hz, 4-H), δ 7.77 (1H, d, ^{3}J = 8.00 Hz,7-H), δ 7.16 (1H, dd, ^{3}J = 8.00 Hz, ^{4}J = 1.60 Hz, 6-H), δ 5.76 (1H, br, s, OH), δ 4.78 (2H, s, CH_2), δ 3.91 (3H, s, N-CH_3); ^{13}C-NMR (DMSO-d_6) δ 153.31 (C-2), δ 142.52 (C-3a), δ 140.89 (C-7a), δ 140.49 (C-5), δ 117.80 (C6), δ 115.06 (C-7), δ 110.58 (C-4) δ 56.36 (C-1′) δ 30.45 (N-CH_3). ν_{max}/cm^{-1} (KBr) 3104 (C-H, *sp*^{2}), 2853 (C-H, *sp*^{3}), 1436 and 1347 (C-H, bend), 1531 and 1339 (N-O).

References

1. Phillips, M.A. The formation of 2-substituted benziminazoles. *J. Chem. Soc.* **1928**, *CCCXVII*, 2393–2399.
2. Mohammadpoor-Baltork, I.; Khosropour, A.R.; Hojati, S.F. Mild and efficient synthesis of benzoxazoles, benzothiazoles, benzimidazoles, and oxazolo[4,5-*b*]pyridines catalyzed by Bi(III) salts under solvent-free conditions. *Monatsh. Chem.* **2007**, *138*, 663–667.
3. Tanaka, K.; Ino, M.; Murakami, Y. Cyclization of [(4- or 5-substituted-2-benzimidazolyl)thio]acetic acids. Isolation and identification of two possible isomers of substituted thiazolo[3,2-*a*]benzimidazol-3(2*H*)-one. *Chem. Pharm. Bull.* **1981**, *29*, 1876–86.
4. Liu, J.; Liu, Q.; Xu, W.; Wang, W. Expeditious and efficient synthesis of benzoxazoles, benzothiazoles, benzimidazoles catalyzed by Ga(OTf)$_3$ under solvent-free conditions. *Chin. J. Chem.* **2011**, *29*, 1739–1744.

5. Evans, D.J.; Thimm, H.F.; Coller, B.A.W. Kinetics, stoicheiometry, and mechanism in the bromination of aromatic heterocycles. Part 5. Aqueous bromination of benzimidazole, 1-methylbenzimidazole, and 2-methylbenzimidazole. *J. Chem. Soc.* **1978**, *9*, 865–871.
6. Fisher, E.C.; Joullie, M.M. Preparation of 5(6)-fluorobenzimidazole and 4(7)-fluorobenzimidazole. *J. Org. Chem.* **1958**, *23*, 1944–1946.
7. Elshihawy, H.E.H. Synthesis and Molecular Modelling of Novel Heterocycles as Inhibitors of Methionine Synthase. Ph.D. Thesis, University of Bradford, Bradford, UK, 2008.
8. Cescon, L.A.; Day, A.R. Preparation of some benzimidazolylamino acids. Reactions of amino acids with *o*-phenylenediamines. *J. Org. Chem.* **1962**, *27*, 581–586.
9. Donkor, R.E. Design, Synthesis and Biological Evaluation of Novel Inhibitors of Cobalamin-Dependent Methionine Synthase. Ph.D. Thesis, University of Bradford, Bradford, UK, 2007.
10. Aridoss, G.; Laali, K.K. Building heterocyclic systems with $RC(OR)^{2+}$ carbocations in recyclable bronsted acidic ionic liquids: Facile synthesis of 1-substituted 1*H*-1,2,3,4-tetrazoles, benzazoles and other ring systems with $CH(OEt)_3$ and $EtC(OEt)_3$ in $[EtNH_3][NO_3]$ and $[PMIM(SO_3H)][OTf]$. *Eur. J. Org. Chem.* **2011**, *15*, 2827–2835.
11. Willitzer, H.; Braeuniger, H.; Engelmann, D.; Krebs, D.; Ozegowski, W.; Tonew, M. Synthesis and antiviral activity of substituted 5-ureido- and 5-thioureidobenzimidazole derivatives. *Pharmazie* **1978**, *33*, 30–38.
12. Gowda, J.; Khader, A.M.A.; Kalluraya, B.; Hidayathulla, S. Synthesis, characterization and antibacterial activity of benzimidazole derivatives carrying quinoline moiety. *Indian J. Chem. Part B Org. Incl. Chem.* **2011**, *50*, 1491–1495.
13. Lahlou, M.; Weliou, M.; Salem, M.; Hajji, M.S. Synthesis and insecticidal activity of some benzimidazolic and benzothiazolic derivatives. *Ann. Pharm. Fr.* **2003**, *61*, 57–61.
14. Grimaldi, A.M.; Day, A.R. Synthesis of Benzimidazolymethyl Analogs of Thiamine. *J. Org. Chem.* **1962**, *27*, 227–229.
15. Khan, M.K.A.; Mohammady, A.; Ahmad, F.Y. Studies on heterocyclics. *Pak. J. Sci. Ind. Res.* **1972**, *15*, 11–12.
16. Wagner-Jauregg, T.; Fischer, J.; Demolis, A. 5,6-Dichloro-2-hydroxymethyl-benzimidazole (DHB), a vitamin B 6 antagonist and synergist. *Arzneimittelforschung* **1972**, *22*, 1324–1328.
17. Rangarajan, M.; Kim, J.S.; Jin, S.; Sim, S.P.; Liu, A.; Pilch, D.S.; Liu, L.F.; LaVoie, E.J. 2″-Substituted 5-Phenylterbenzimidazoles as Topoisomerase I Poisons. *Bioorg. Med. Chem.* **2000**, *8*, 1371–1382.
18. King, F.E.; Acheson, R.M.; Spensley, P.C. 298. Benziminazoles related to pteroic and pteroylglutamic acids. *J. Chem. Soc.* **1949**, 1401–1406, doi:10.1039/JR9490001401.
19. Dandegaonker, S.H.; Revankar, G.R. Brom-hydroxychalkone. *Monatsh. Chem. Wiss.* **1965**, *96*, 450–460.
20. Labas, R.; Gilbert, G.; Nicole, O.; Dhilly, M.; Abbas, A.; Tirel, O.; Buisson, A.; Henry, J.; Barre, L.; Debruyne, D.; *et al.* Synthesis, evaluation and metabolic studies of radiotracers containing a 4-(4-[18F]-fluorobenzyl)piperidin-1-yl moiety for the PET imaging of NR2B NMDA receptors. *Eur. J. Med. Chem.* **2011**, *46*, 2295–2309.

21. Prostakov, N.S.; Varlamov, A.V.; Nende, D.L.S.; Krapivko, A.P.; Fomichev, A.A.; Golovtsov, N.I.; Shendrik, I.V.; Aliev, A.E.; Singh, B.B. Pyrido[1,2-a]benzimidazole reactions. *Zh. Org. Khim.* **1990**, *26*, 1351–1356.
22. Kempe, U.; Dockner, T.; Koehler, H. 1989. Preparation of Imidazole-2-carboxylic Acids. Application: DEDE Patent Application. Available online: http://patents.justia.com/patent/5117004 (accessed on 14 August 2015).
23. Thakurdesai, P.A.; Wadodkar, S.G.; Chopade, C.T. Synthesis and anti-inflammatory activity of some benzimidazole-2-carboxylic acids. *Pharmacologyonline* **2007**, *1*, 314–329.
24. Kurihara, T.; Ro, K.; Katsuyama, M. Benzimidazolecarboxylic acid derivatives. *Tohoku Yakka Daigaku Kiyo* **1960**. 7, 95–102.
25. Bednyagina, N.P.; Postovskii, I.Y. Hydrolytic cleavage of some sulfones of heterocyclic series. VI. Synthesis and properties of *p*-nitrophenylsulfonyl-*N*-methylbenzimidazolyl methane and *p*-nitrophenylsulfonyl benzothiazolyl methane. *J. Gen. Chem. U.S.S.R. Eng. Transl.* **1960**, *30*, 3193–3196.
26. Popov, I.I.; Simonov, A.M.; Zubenko, A.A. Synthesis of 2-vinylbenzimidazole. *Chem. Heterocycl. Compd.* **1976**, 12, 946.
27. Khristich, B.I.; Tsupak, E.B.; Kononogova, O.I. Free-radical hydroxymethylation of benzimidazole. *Chem. Heterocycl. Compd.* **1982**, *18*, 1299–1302.
28. Harisha, R.S.; Hosamani, K.M.; Keri, R.S. Synthesis, *in vitro* microbial and cytotoxic studies of new benzimidazole derivatives. *Arch. Pharm.* **2009**, *342*, 412–419.
29. Dellweg, H.; Becher, E.; Bernhauer, K. Biosynthesis in the cobalamin series. I. Cobalamin analogs of the benzimidazole series. *Biochem. Z.* **1956**, *327*, 422–449.
30. Cowart, M.; Latshaw, S.P.; Bhatia, P.; Daanen, J.F.; Rohde, J.; Nelson, S.L.; Patel, M.; Kolasa, T.; Nakane, M.; Uchic, M.E.; *et al.* Discovery of 2-(4-pyridin-2-ylpiperazin-1-ylmethyl)-1*H*-benzimidazole (ABT-724), a dopaminergic agent with a novel mode of action for the potential treatment of erectile dysfunction. *J. Med. Chem.* **2004**, *47*, 3853–3864.
31. Ishii, T.; Sugane, T.; Munakata, R.; Aoki, S.; Higaki, M.; Someya, A. Carbamate Compound or Salt Thereof. Astellas Pharma Inc. Patent No. 2008–285466, 2010. Available online: http://patentscope.wipo.int/search/en/detail.jsf?docId=WO2010053120&recNum=217&tab=PCTDocuments&maxRec=388&office=&prevFilter=&sortOption=&queryString=PA%2F%22Astellas%22 (accessed on 22 June 2015).
